# 2554. What’s Weight Got to Do with It? Prospective Pharmacokinetic/Pharmacodynamic Evaluation of Fixed-Dose Daptomycin

**DOI:** 10.1093/ofid/ofad500.2171

**Published:** 2023-11-27

**Authors:** Katie B Olney, Jenni Thomas, Manjunath P Pai, Donna R Burgess, William Olney, Rebecca Bruning, Kamron Amir Griffith, Danielle Casaus, Elizabeth Crance, David Burgess

**Affiliations:** University of Kentucky HealthCare, Lexington, Kentucky; University of Virginia Health, Charlottesville, Virginia; University of Michigan, Ann Arbor, Michigan; UK HealthCare, Lexington, KY; University of Kentucky HealthCare, Lexington, Kentucky; University of Kentucky HealthCare, Lexington, Kentucky; Parkland Health, Dallas, Texas; University of Kentucky HealthCare, Lexington, Kentucky; University of Kentucky HealthCare, Lexington, Kentucky; UK HealthCare, Lexington, KY

## Abstract

**Background:**

As daptomycin (DAP) CL does not scale proportionally with weight, fixed-dose DAP has been proposed to optimize efficacy and mitigate toxicity. This study evaluated systemic exposure of a fixed dose (750 mg) of DAP in patients with *Staphylococcus aureus* infections.

**Methods:**

Adult non-ICU patients with CrCl ≥ 30 mL/min admitted to our academic medical center warranting DAP for a *S. aureus* infection were eligible for enrollment. DAP 750 mg was infused over 30 minutes every 24 hours. At steady state, concentrations were obtained pre-dose (0.5 hours) and 1, 12, and 24 hours after the start of infusion. Concentrations were measured using a validated LC-MS assay. Pharmacokinetic (PK) analysis was performed using first-order equations and stratified by weight: low (< 65 kg), normal (65-85 kg), and high ( > 85 kg). Therapeutic AUC_24_ (≥ 666 mg*hr/L) and elevated trough (C_min_ > 24.3 mg/L) were the efficacy and safety targets, respectively.

**Results:**

Overall, 22 patients (6 low weight, 8 normal weight, 8 high weight) were enrolled with no differences observed in baseline characteristics (median [IQR] age 49 [42.2, 56.5], CrCl 111 mL/min [79, 134]). PK parameters (median [IQR]) did not differ by weight with Vd 9.9 L [6.3, 17.2], K_e_ 0.078 h^-1^ [0.046, 0.098], CL 0.87 L/h [0.47, 1.34], AUC_24_ 861 mg*hr/L [538, 1541], and C_min_ 10.5 mg/L [7.3, 29.9]. Therapeutic AUC_24_ was achieved in 14/22 (64%) and elevated C_min_ occurred in 7/22 (32%), with no differences seen among weight groups. However, differences were noted between males (n=10) and females (n=12) with significantly higher CL (median [IQR]) noted in males (1.26 L/h [0.92, 1.48]) vs. females (0.49 L/h [0.37, 0.86]) (*p*=0.027). Hence, median [IQR] AUC_24_ was significantly lower in males at 594 mg*hr/L [454, 736] vs. 1513 mg*hr/L [856, 1879] in females (*p*=0.021). Therapeutic AUC_24_ was achieved in 40% of males versus 83% of females, while elevated C_min_ was seen in 20% of males and 32% of females.

**Conclusion:**

DAP PK and exposure (AUC_24_ and C_min_) was not impacted by weight. These results support fixed, rather than weight-based, dosing of DAP in non-ICU patients with normal renal function. Notably, gender differences were observed with males having significantly higher CL, likely warranting higher doses of DAP to ensure optimal exposure.

**Disclosures:**

**Katie B. Olney, PharmD, BCIDP**, The Society of Infectious Diseases Pharmacists (SIDP): Grant/Research Support

